# Editorial: Adverse outcomes of preeclampsia: from mother to baby, pregnancy to postpartum

**DOI:** 10.3389/fphys.2024.1394120

**Published:** 2024-04-08

**Authors:** Bhavisha A. Bakrania, Frank T. Spradley, Lana McClements

**Affiliations:** ^1^ UQ Centre for Clinical Research and Perinatal Research Centre, The University of Queensland, Brisbane, QLD, Australia; ^2^ Department of Surgery, University of Mississippi Medical Center, Jackson, MS, United States; ^3^ Department of Pharmacology and Toxicology, University of Mississippi Medical Center, Jackson, MS, United States; ^4^ Department of Physiology and Biophysics, University of Mississippi Medical Center, Jackson, MS, United States; ^5^ School of Life Sciences and Institute for Biomedical Materials and Devices, University of Technology Sydney, Sydney, NSW, Australia

**Keywords:** preeclampsia, biomarkers, neurodevelopment, women’s health, post-partum, placenta

Preeclampsia is a dangerous pregnancy-specific complication that involves new-onset hypertension and signs of systemic vascular dysfunction (Bisson et al.). Beyond pregnancy, women are at greater risk for devastating illnesses including cardiac disease (deMartelly et al., 2021), Alzheimer’s disease (Theilen et al., 2016) and stroke (Bellamy et al., 2007; Theilen et al., 2016). Moreover, their offspring present with disturbances in hormonal, metabolic and neurodevelopmental pathways that likely accelerate onset of disease with age (Nahum Sacks et al., 2018; Yang et al., 2023). These observations warrant ongoing research, not only to understand the mechanisms, but because clinicians lack reliable pharmacotherapies to cure preeclampsia. In this editorial, we present pre/clinical original research and literature reviews, including work on biomarkers and molecular pathways that are necessary to identify druggable targets to halt sudden and long-term ramifications.

Preeclampsia has been recognized to foreshadow ensuing chronic disease in women and their baby, which demonstrates the need for advances in earlier prediction and interventions. On this Research Topic, Bisson et al. reviewed a set of studies stressing the requirement to include biomarkers beyond gestational-related blood pressure levels as the latter alone does not strongly predict preeclampsia. Work from this team and their colleagues has contributed to our understanding of the powerful predictive nature of sFlt-1:PlGF ratios in preeclampsia (Bisson et al.) whereby elevations are indicative of a vasoconstrictive milieu within the maternal circulation. Combining this value with blood pressure has a strong association with hospitalizations due to preeclampsia. It is fathomable that strategies to confidently predict preeclampsia as early as possible would allow time to combat and prevent this disease.

Han et al. reviewed use of biomarkers for predicting late-onset, term preeclampsia, which is more prevalent and not as well detected as early-onset preeclampsia. They found that there are currently no molecular biomarkers with sufficient clinical sensitivity and specificity. Several limitations likely muddled these results but could be overcome standardizing definitions of preeclampsia subtypes as well as sample collection. In a study by Kandel et al. that assessed biomarkers for early- and late-onset preeclampsia, they found reduced galectin-3, which is a marker of cardiovascular disease and heart failure (Chen et al., 2021) in non-pregnant patients, in the former and not latter subtype. However, in another study where preeclampsia subtypes were combined, expression of galectin-3 was increased (Ghorbanpour et al., 2023). Thus, more work is needed to determine if there are biomarkers that can predict timing and severity of preeclampsia.

While biomarkers help to educate clinicians on management options, there are educational opportunities for the patient that could emphasize the seriousness of hypertension in preeclampsia that are associated with lower blood pressure after pregnancy, including: 1) awareness that adversities from preeclampsia can extend past parturition, such as cardiac abnormalities (deMartelly et al., 2021), and dictate a future shortened by hypertensive disease in mother and baby and 2) programs that simplify adoption of routine blood pressure measurement and follow-up appointments. Added opportunities to seek advice, such as with telehealth medicine, are likely needed as online sentiments of emotional support increase and stay high during pregnancy (Hou and Hou).

Beckett et al. reviewed cerebrovascular disease and cognitive impairment in preeclampsia and beyond. The psychological outcomes of pregnancy can be adversely influenced by preeclampsia targeting the cerebrovasculature. Importantly, preeclampsia can quickly advance to eclampsia, which manifests as new-onset seizures and accounts for 13% of maternal deaths worldwide (Nour, 2008; Beckett et al.). Although not fully understood, one mediator may be white matter lesions, which are present in over 60% of women with a history of preeclampsia (Soma-Pillay et al., 2017). Another possible culprit is inflammation. Herrock et al. described how pro-inflammatory cells and cytokines cause widespread endothelial injury. This can disrupt the blood brain barrier and feedforward to promote neuroinflammation, cerebrovascular dysfunction, and white matter lesioning (Beckett et al.).

Neurodevelopmental disorders occur at a greater rate in offspring born from preeclamptic pregnancies. Several studies report that they have increased risk of autism spectrum disorder, attention deficit/hyperactivity disorder and intellectual disability (Ehrenstein et al., 2009; Dang et al., 2016; Alsnes et al., 2017; Nahum Sacks et al., 2018; Gumusoglu et al., 2020). Although these associations exist, less is known about the pathogenic mechanisms. Interluekin-6 (IL-6) is elevated in preeclampsia and contributes to the pro-inflammatory environment that exists in this disease (Bakrania et al., 2020). Despite this, Barron et al. found that in neuroblastoma cells exposed to sera from preeclamptic women, neurite growth and mitochondrial respiration were elevated, in part, due to increased IL-6. Further exploration of the neurodevelopmental implications of this, and other pathogenic factors are critical to properly understand underlying mechanisms.

There is also increased risk for systemic cardiovascular, metabolic and reproductive diseases in preeclampsia-exposed offspring. In a preclinical study, the timing of onset for puberty was significantly earlier in female, but not male, mice originating from preeclamptic BPH/5 mice (Gomes et al.). These data coincide with previous literature that indicate a dominant androgenic profile in female offspring of pregnancies affected by preeclampsia (Ogland et al., 2011; Alsnes et al., 2016). Cardiometabolic characteristics, that are also associated with increased risk of preeclampsia, have also been documented to be elevated in female offspring of BHP/5 mice, but not their male littermates, such as increased food intake, increased body weight, hyperleptinaemia and increased visceral white adipose tissue. Preclinical studies show that overexpression of leptin promotes the pathogenesis of this disease and continued research focuses on whether elevated levels are a novel biomarker for preeclampsia (Wang et al.).

In summary, our editorial highlights original research and literature reviews that focus on the sequelae of preeclampsia. Such adversities impact both mothers and their offspring, not only during, but well beyond pregnancy. There is a desperate need for translational research that utilize human data on biomarkers to design preclinical models assessing their pathogenic potential as targets for pharmacotherapies to prevent outcomes of preeclampsia. Aside from finding a cure, recommendations include a focus on standardizing diagnostic criteria and programs to support *postpartum* care of women and their children.
